# Heterotopic adrenal tissue in a specimen after unilateral salpingo-oophorectomy: a case report

**DOI:** 10.1186/s13256-025-05290-3

**Published:** 2025-05-14

**Authors:** Dominika Trojnarska, Ewa Zabiegło, Robert Jach

**Affiliations:** 1 Faculty of Health Sciences, Jagiellonian University Medical College, Kraków, Poland; 2 Faculty of Medicine, Jagiellonian University Medical College, Kraków, Poland

**Keywords:** Heterotopic adrenal tissue, Adult, Salpingo-oophorectomy, Case report

## Abstract

**Background:**

Heterotopic adrenal tissue is an extremely rare finding. The most common site is the genitourinary tract and pelvis, more frequently in male than female children. In our report, we discuss an ectopic adrenal tissue detected incidentally in a perimenopausal woman, which is even more unusual.

**Case presentation:**

A 54-year-old Eastern European perimenopausal female patient was referred for surgical treatment due to a suspected 60 mm paratubal cyst. Her medical history was unremarkable and risk of ovarian malignancy algorithm score were normal. Laparoscopic left salpingo-oophorectomy was performed and the specimen was sent for histopathology. The examination revealed a normal ovary, a fallopian tube with a simple paratubal cyst, and a 2.5 mm nest of heterotopic adrenal tissue in the nearby fat tissue. The patient was discharged on the first postoperative day, reported no symptoms, and remained asymptomatic at 3-week follow-up.

**Conclusion:**

A review of the available English literature confirmed the rarity of heterotopic adrenal tissue in adult women. This case is presented due to its uniqueness, with the aim of raising awareness about the entity we encountered and presenting its possible implications.

## Background

Heterotopic adrenal tissue, first discovered by Morgagni in 1740, is a rare condition that can be located in various sites, typically within the retroperitoneum or pelvis along the path of gonadal descent [1, 2]. The exact prevalence of this condition is unknown, but is considered to be found predominantly in the male pediatric population and in less than 1% of adults [3]. The adrenal rests are usually clinically silent and are often discovered incidentally in surgical specimens. Still, it is important to consider hormonal evaluation in such cases, particularly in the context of potential adrenal insufficiency or hormone-producing ectopic tissue.

In this article, we report a case of an ectopic adrenal rest in a 54-year-old female patient, which was discovered incidentally during a routine pathologic evaluation of a salpingo-oophorectomy specimen, surgically removed on the grounds of paratubal cyst.

## Case presentation

A 54-year-old Eastern European perimenopausal female patient was referred to our hospital for surgical treatment due to a suspected 60 mm paratubal cyst with a tendency to grow. Her personal medical history included primary hypertension, hypercholesterolemia, and impaired glucose tolerance; her blood pressure was normotonic, HbA1c levels were within normal range, and only a slight elevation in low-density lipoprotein (LDL) cholesterol was observed. She had previously undergone dilation and curettage for abnormal uterine bleeding a couple of years earlier and was since then asymptomatic. The gynecological examination was unremarkable, and routine preoperative blood tests, including complete blood count, activated partial thromboplastin time (aPTT), prothrombin time (PT), sodium, and potassium levels, were within normal limits. The risk of ovarian malignancy algorithm score (ROMA) score was low.

**Fig. 1:**
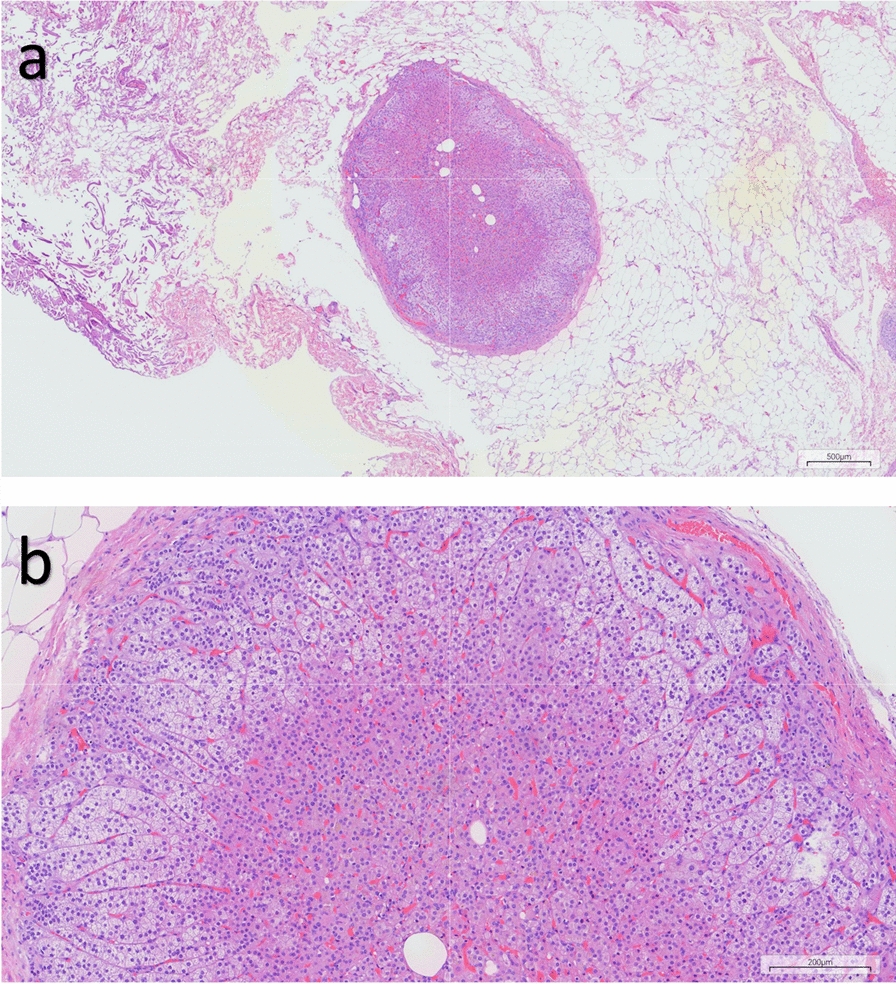
Adrenal ectopia within the adipose tissue near the fallopian tube. Hematoxylin and eosin staining show a well-defined nodule composed of cortical cells only and with a thin capsule in the adipose tissue. (**a**) Scale bar indicates 500 μm (× 40 magnification); (**b**) scale bar indicates 200 μm (× 100 magnification)

On the basis of clinical and ultrasonographic assessment, a laparoscopic left salpingo-oophorectomy was performed, and the excised specimen was submitted for histopathological examination. The analysis revealed an ovary measuring 18 mm in diameter with preserved architecture, including corpora albicantia, as well as a structurally intact fallopian tube. A simple paratubal cyst was identified adjacent to the fallopian tube wall. Additionally, a 2.5 mm focus of heterotopic adrenal tissue composed of normal adrenal cortex was detected within the adipose tissue near the fallopian tube (Fig. 1).

The patient was released on the first postoperative day and reported for a follow-up visit 3 weeks after the surgery. She was asymptomatic. The physical exam did not show any abnormalities. The blood tests, including adrenal-specific laboratory tests—adrenocortical hormone (ACTH), cortisol, dehydroepiandrosterone sulfate (DHEAS), and aldosterone—were ordered. All results were within normal limits, with no evidence of hormonal excess or insufficiency. Additionally, the patient’s comorbidities, including hypertension and glucose intolerance, remained unchanged postoperatively.

## Discussion

Heterotopic adrenal tissue, also known as ectopic adrenal tissue or adrenal rests, represents nests or foci of adrenal tissue detected in various locations distant from the eutopic adrenal glands [4]. They are frequently detected in retroperitoneal, pelvic, or groin areas, mainly composed of adrenocortical component and can be detected in both pediatric, and less often, adult patients [2, 5, 6]. The most commonly affected anatomical region, accounting for approximately 50% of cases, includes the paraovarian, ovarian, parasalpingeal areas, the infundibulopelvic ligament, and the broad ligament. Approximately 30% are identified in the spermatic cord and paratesticular regions. Inguinal hernia sacs and inguinal fat account for an additional 15% of cases. The remaining 5% are distributed across less common sites, including the peritoneum, appendiceal mesentery, and rare intraabdominal locations such as intrahepatic and intrarenal regions [5, 7]. Common sites are considered to be due to abnormalities of fusion or persistent remnants of the adrenal ridge, while rare sites could be explained by abnormal migration of adrenal tissue during gestation or differentiation of adrenal progenitor stem cells [6, 8].

The adrenal gland has a dual embryological origin, comprising a cortex derived from the intermediate mesoderm and a medulla originating from neural crest cells. Adrenal rest tissue may consist of cortical components alone or both cortical and medullary elements, depending on whether separation occurs before or after the migration of medullary tissue into the developing cortex [9]. Notably, during embryogenesis, the adrenal cortex maintains a close anatomical relationship with the developing gonads, which may contribute to the presence of ectopic adrenal tissue along the path of gonadal descent [9, 10].

Ectopic adrenal tissue typically presents as a single nodule and rarely as multiple nodules, usually measuring less than 10 mm in greatest dimension, as in this case [7]. Generally, these nodules are endocrinologically inactive and are usually discovered incidentally during surgery or microscopic evaluation [3, 4, 6]. However, hyperplastic ectopic adrenal glands are often found in cases of congenital adrenal hyperplasia due to chronic ACTH stimulation [11]. They may become clinically symptomatic as a result of hormonal activity, mass effect, or a combination of both [11, 12].

Diagnosis of adrenal rests is typically made through histopathological examination, often as an incidental finding, since the lesion is usually hormonally silent. There are no characteristic laboratory findings associated with ectopic adrenal tissue. However, it may undergo hyperplastic changes in response to elevated levels of ACTH, as observed in conditions such as Cushing’s disease or Nelson’s syndrome [3, 7, 13]. In some cases, hypercortisolism may develop in the form of ACTH-independent Cushing’s syndrome, presenting with clinical features such as hypertension, fasciotruncal obesity, and virilization [14, 15]. In rare cases, heterotopic adrenal tissue may undergo malignant transformation, similar to that observed in the normal adrenal gland [4, 7]. Radiologically, these lesions are generally undetectable. Only in rare instances may a mass be visualized if an adrenocortical tumor arises from the ectopic tissue [3, 7].

Treatment is generally not required unless a neoplasm develops within the tissue or the patient becomes symptomatic, most commonly due to adenomas, and in exceptionally rare cases, carcinomas [3, 7, 16, 17]. Some authors suggest that ectopic adrenal tissue should always be excised if incidentally encountered during surgery [18].

The differential diagnosis for heterotopic adrenal tissue includes testicular or ovarian Sertoli–Leydig cell tumor, primary or metastatic clear cell renal cell carcinoma, hilus cells, paraganglioma or ganglion with clear cell change, and metastatic adrenal cortical carcinoma [5].

The clinical approach presented in this case highlights the use of additional diagnostic tools that were guided by the histopathological findings, including adrenal-specific laboratory testing and monitoring of preexisting conditions that could potentially be influenced by the ectopic adrenal tissue. However, as the majority of patients remain asymptomatic, even extensive laboratory testing often yields unremarkable results.

## Conclusion

Heterotopic adrenal tissue in the adult population constitutes a rare entity, especially among women. The lesions are mostly asymptomatic and revealed incidentally during surgery or histopathological examination. Despite limited clinical implications, it is essential to be aware of the possibility of adrenal rests in adults, as the ectopic tissue may develop the same pathologies as the normal adrenal gland.

## Data Availability

Any additional data is available upon request.

## Abbreviations

17-OHP: 17-Hydroxyprogesterone
ACTH: Adrenocortical hormone
ROMA: Risk of ovarian malignancy algorithm score
